# Inhibition of myeloperoxidase oxidant production by *N*-acetyl lysyltyrosylcysteine amide reduces brain damage in a murine model of stroke

**DOI:** 10.1186/s12974-016-0583-x

**Published:** 2016-05-24

**Authors:** Guoliang Yu, Ye Liang, Ziming Huang, Deron W. Jones, Kirkwood A. Pritchard, Hao Zhang

**Affiliations:** Division of Pediatric Surgery, Department of Surgery, Medical College of Wisconsin, 8701 Watertown Plank Rd., Milwaukee, WI 53226 USA; Department of Breast Surgery, Maternal and Child Health Hospital of Hubei Province, 745 WuLuo Road, Hongshan District, Wuhan City, Hubei Province 430070 China

**Keywords:** Stroke, Myeloperoxidase, Oxidative stress, *N*-acetyl lysyltyrosylcysteine amide (KYC), Middle cerebral artery occlusion (MCAO)

## Abstract

**Background:**

Oxidative stress plays an important and causal role in the mechanisms by which ischemia/reperfusion (I/R) injury increases brain damage after stroke. Accordingly, reducing oxidative stress has been proposed as a therapeutic strategy for limiting damage in the brain after stroke. Myeloperoxidase (MPO) is a highly potent oxidative enzyme that is capable of inducing both oxidative and nitrosative stress in vivo.

**Methods:**

To determine if and the extent to which MPO-generated oxidants contribute to brain I/R injury, we treated mice subjected to middle cerebral artery occlusion (MCAO) with *N*-acetyl lysyltyrosylcysteine amide (KYC), a novel, specific and non-toxic inhibitor of MPO. Behavioral testing, ischemic damage, blood-brain-barrier disruption, apoptosis, neutrophils infiltration, microglia/macrophage activation, and MPO oxidation were analyzed within a 7-day period after MCAO.

**Results:**

Our studies show that KYC treatment significantly reduces neurological severity scores, infarct size, IgG extravasation, neutrophil infiltration, loss of neurons, apoptosis, and microglia/macrophage activation in the brains of MCAO mice. Immunofluorescence studies show that KYC treatment reduces the formation of chlorotyrosine (ClTyr), a fingerprint biomarker of MPO oxidation, nitrotyrosine (NO_2_Tyr), and 4-hydroxynonenal (4HNE) in MCAO mice. All oxidative products colocalized with MPO in the infarcted brains, suggesting that MPO-generated oxidants are involved in forming the oxidative products.

**Conclusions:**

MPO-generated oxidants play detrimental roles in causing brain damage after stroke which is effectively reduced by KYC.

**Electronic supplementary material:**

The online version of this article (doi:10.1186/s12974-016-0583-x) contains supplementary material, which is available to authorized users.

## Background

Oxidative stress is considered as an important, causal factor in the mechanisms by which stroke induces brain injury [1, 2]. Extensive studies have shown that the increase in oxidative stress induced by I/R after acute stroke plays a critical role in brain tissue injury. Immediately after acute ischemic stroke, reactive oxygen species (ROS) and reactive nitrogen species (RNS) production increases rapidly, resulting in severe damage to ischemic tissues. Moreover, restoring blood flow to ischemic tissue (reperfusion) induces even greater increases in ROS/RNS production [3], which can induce more severe tissue injury. Production of ROS, such as hydroxyl radicals [4], superoxide (O_2_^−●^) [5], peroxynitrite (ONOO^−^) [6], and hydrogen peroxide (H_2_O_2_) [7], have all been reported to be increased in animal models of stroke. Consequently, oxidation products are increased in stroke models [8]. Moreover, plasma levels of protein carbonyls are increased in stroke patients as well as lipid peroxidation products [9, 10]. Importantly, plasma malondialdehyde (MDA) levels in stroke patients appear to correlate with their stroke severity and clinical outcomes [11, 12]. Taken together, these reports strongly support the idea that ROS and RNS play important roles in the mechanisms by which stroke induces and propagates tissue injury and brain cell death after stroke.

Although a considerable amount of evidence exists for oxidative stress inducing neuronal damage in stroke, clinical trials have failed to show that antioxidants significantly improve outcomes [13–15]. Exactly why is still unclear although several reasons have been proposed (for details see [13–15]). One of the conclusions from those studies is that agents targeting specific sources of oxidative stress may be more effective for therapy for stroke than a general antioxidant.

Until recently, the role of myeloperoxidase (MPO) in stroke has been limited to serving as a biomarker for neutrophil infiltration. MPO is a highly versatile oxidative enzyme, capable of inducing both oxidative and nitrosative stress in vivo [16]. After activation with H_2_O_2_, MPO oxidizes substrates (chloride (Cl¯), bromide (Br¯), nitrite (NO_2_¯), tyrosine (Tyr), etc.) to potent oxidants (hypochlorous acid (HOCl) or hypobromous acid (HOBr)) and free radicals (nitrogen dioxide (^●^NO_2_) or tyrosyl radical (Tyr^●^) etc.), respectively. These free radicals and oxidants are more potent than O_2_^−●^ and H_2_O_2_ for oxidizing biomolecules and inducing cellular injury [16]. Although MPO is rapidly released from activated neutrophils, monocytes, and some macrophages upon activation [17], MPO is also expressed by activated microglia, astrocytes, and certain types of neurons in neurodegenerative disease [18–22].

In stroke, tissue MPO levels are routinely used to assess neutrophil infiltration [23]. However, recent studies suggest that MPO plays a detrimental role in stroke via its ability to generate highly reactive oxidants and toxic free radicals. Support for this idea comes from studies showing that serum MPO levels are elevated after acute stroke [24, 25]. Further, increased serum MPO levels in stroke patients have been associated with white matter hyperintensity, a measure of stroke severity assessed from brain MRI scans [26]. MPO has been suggested as a biomarker for diagnosis and prognosis of stroke [27]. A recent report showed that 4-aminobenzoic acid hydrazide (ABAH), a classic MPO inhibitor, reduced infarct size and neuronal deficit in middle cerebral artery occlusion (MCAO) mice [28]. Taken together, these reports provide strong evidence for direct links between MPO activity and severity of brain injury in stroke, providing the rationale for inhibiting MPO activity as a novel therapeutic strategy for stroke.

Recently, we developed a new MPO inhibitor, *N*-acetyl lysyltyrosylcysteine amide (KYC), and demonstrated that it is a potent, reversible, specific, and non-toxic inhibitor of MPO [29]. Specificity of KYC for inhibition of MPO has been extensively verified in in vitro, cell models and animal models [29, 30]. Most classic MPO inhibitors suffer high toxicity either by inherent toxicity or yielding harmful secondary radicals after inhibition that cause cell injury and death (for review see [31] and [32]). However, when KYC inhibits MPO activity, MPO can oxidize tyrosine to a potent tyrosyl radical that is rapidly scavenged by nearby cysteine to form a thiyl radical that results in the formation of disulfides. Thus, the unique design of KYC ensures no cell injury caused by harmful secondary radicals formed during inhibition [29]. KYC inhibition of MPO has been shown to improve vasodilatation in sickle cell disease mice [33] and even inhibit tumor formation in a neutrophil-dependent solid tumor, methylcholanthrene-initiated, butylated hydroxytoluene-promoted murine model of lung cancer [34]. More recently, we reported that KYC reduces disease score severity in two distinct murine models of experimental autoimmune encephalomyelitis (EAE) [30]. These studies show that KYC not only decreased oxidized proteins and MPO levels but also restored blood-brain-barrier (BBB) function and decreased neutrophil infiltration in the central nervous system (CNS) of EAE mice [30]. Importantly, KYC did not reduce disease scores in MPO knockout EAE mice, demonstrating that KYC specifically targets MPO in vivo [30]. Taken together, our findings suggest that KYC is a specific, effective, and non-toxic MPO inhibitor that is capable of reducing MPO-dependent oxidative stress in the CNS. In the present study, we investigate if KYC reduces brain injury in MCAO mice and the extent to which MPO-dependent oxidative stress mediates brain injury and cell death after stroke.

## Methods

### Animal model of focal cerebral ischemia

C57BL/6J mice (8–10 weeks old) were purchased from the Jackson Laboratory (Bar Harbor, ME). All mice were housed in the Medical College of Wisconsin, with 12-h light/dark cycle and allowed free access to food and water. All animal procedures were approved by the Institutional Animal Care and Use Committee. Animals were anesthetized with 2 % isoflurane. A rectal temperature probe was inserted to monitor and maintain a constant animal core temperature of 37 ± 0.5 °C using a temperature controller (TC-1000, CWE INC, Ardmore, PA). Transient focal cerebral ischemia was induced by middle cerebral artery occlusion (MCAO) as described by Li et al. [35]. Briefly, a 6–0 nylon monofilament suture coated with silicon-rubber (Doccol, Sharon, MA) was inserted into the left internal carotid artery and advanced approximately 10 mm distal to the carotid bifurcation to occlude the origin of the middle cerebral artery. The thread was carefully withdrawn 30 min after MCAO to induce I/R injury. In sham-operated animals, the same procedure was done with the exception of inserting the intraluminal filament. During first 3 days after ischemia, the body temperature of animals was maintained using a heating pad with Gaymar T/pump (Stryker Inc., Kalamazoo, MI). The temperature was set at 37 °C.

### Neurobehavioral testing

Neurologic severity scores were determined by a number of tests to assess motor, sensory, and reflex [35]. Briefly, after raising the mouse by the tail, flexion of forelimb, head movement >10° to vertical axis, and circling toward paralytic side were assessed. Three more tests were performed by placing the mouse on the floor to assess abnormal gait, circling toward the paralytic side, and frequency of falling over. Finally, pinna reflex (a head shake upon touching the auditory meatus) and visual placement test (stretching of forelimbs to meet an approaching object) were also evaluated. Each test was scored as 0 for normal and 1 for abnormal, yielding a summed injury score from 0 to 8.

### Drug administration

Groups of mice were administered either phosphate-buffered saline (PBS) or KYC (Biomatik, Wilmington, Delaware) 10.0 mg/kg daily intraperitoneally started from 1 h before or 1 h after MCAO. Mice were treated daily for 3 or 7 days after MCAO. The dosage of KYC was determined according to the pharmacokinetics of KYC in plasma published earlier [33] and our previous study on the effects of KYC in a murine EAE model of multiple sclerosis [32].

### Histopathology

Three days after ischemia, mice were anesthetized and perfused transcardially with 4 % paraformaldehyde after pre-washing with 0.01 M PBS. Brain tissues were then fixed in 4 % paraformaldehyde overnight and were transferred to 20 and 30 % sucrose for 1 day, respectively. Six serial coronal slices were prepared at 1-mm intervals from the frontal pole. Sections were cut by cryomicrotomy (CM1900, Leica, Germany); 10-μm sections were used for immunohistochemistry and 20-μm sections were prepared for histological staining with 0.5 % cresyl violet [36]. The infarct area of brain tissue was defined as the area showing reduced cresyl violet staining. Data were confirmed by light microscopy using dark pyknotic-necrotic cell bodies. The areas of infarct and both hemispheres of each brain section were determined using a National Institutes of Health (NIH) ImageJ. To partially correct for effects of edema, the corrected infarct area was determined as described by Swanson et al. [37]: RT-LN, where RT = total area of the right non-ischemic hemisphere, and LN = non-infarcted area in the left ischemic hemisphere of the same section. Lesion volume of each section was calculated as corrected lesion area × slice thickness (1 mm). The total lesion volume was the summation of the lesion volumes of all brain sections.

### Immunohistochemistry

The following protocol was used to determine neutrophil accumulation, microglia/macrophage activation, p53 expression, and neuron density in the ischemic core of the brain cortex. Frozen sections (10 μm) were incubated with 5 % goat or donkey serum in 0.01 M PBS for 1 h. The sections were incubated with rat anti-NIMP-R14 (Abcam, Cambridge, MA; 1:50; marker of neutrophils), goat anti-Iba1 (Abcam; 1:200; marker of microglia/macrophages), mouse anti-p53 (Abcam; 1:50), or mouse anti-NeuN (Abcam, 1:50; marker of neuron) antibodies at 4 °C, overnight. The next day, sections were rinsed and then incubated with secondary antibodies conjugated with Alexa Fluor 568 (1:200) for 1 h. Finally, the sections were counterstained with DAPI to visualize cell nuclei. To determine ClTyr or NO_2_Tyr accumulation and colocalization with MPO, brain sections were doubly immunostained overnight with rabbit anti-ClTyr (Hycult Biotech, Plymouth Meeting, PA; 1:50) and mouse anti-MPO antibodies (Hycult Biotech; 1:50) or mouse anti-NO_2_Tyr (Santa Cruz, Dallas, TX; 1:50) and rabbit anti-MPO (Abcam; 1:50) antibodies. Next, the sections were incubated with secondary antibodies conjugated with Alexa Fluor 488 or 568, respectively. Comparable brain sections in mice from PBS and KYC groups were selected for analysis. Images of three areas in the cortex from three predetermined corticostriatal sections with largest infarct profiles were captured at random using a fluorescence microscope (DP71, Olympus America Inc., Center Valley, PA). Counting of the immunostained positive cells in each area was determined and calculated as counts per square millimeter by a single “blind” investigator, who had no knowledge of assignment of treatment groups using NIH ImageJ.

Endogenous IgG immunostaining was performed to detect BBB disruption [38]. Brain sections were incubated successively with peroxidase-conjugated anti-mouse IgG antibody (Jackson ImmunoResearch, West Grove, PA; 1:200) and 3,3′-diaminobenzidine. Gray scale in the immunostained sections was quantified using NIH ImageJ. IgG exudation was expressed as a percentage of the increase in gray scale in the ischemic hemisphere: (Gi–G0)/G0 × l00%, where Gi is the gray scale of the ischemic hemisphere and G0 is the gray scale of the contralateral non-ischemic hemisphere.

### Terminal deoxynucleotidyl transferase-mediated dUTP-biotin nick end labeling assay

The terminal deoxynucleotidyl transferase-mediated dUTP-biotin nick end labeling (TUNEL) assay was used to identify apoptotic cells with nuclear DNA fragmentation in brain ischemic core areas. Staining was performed according to the manufacturer’s instructions (Click-iT Plus TUNEL Kit, Thermo Fisher, Waltham, MA). Briefly, brain sections adjacent to those used for immunohistochemistry were incubated with proteinase K (15 min, RT) and then rinsed with PBS. After incubation with TdT reaction buffer (10 min) and TdT reaction mixture (1 h at 37 °C), sections were washed and incubated with Click-iT Plus reaction cocktail containing Alexa Fluor 488 (30 min at 37 °C). Finally, sections were counterstained with DAPI.

### Western blotting

Three days after ischemia, anesthetized mice were perfused with PBS and brain tissues collected and stored at −80 °C. Brain tissue proteins were extracted into radio-immunoprecipitation assay (RIPA) buffer containing Protease Inhibitor Cocktail and ethylenediaminetetraacetic acid (EDTA; Thermo Fisher Scientific, Inc., Waltham, MA; 1: 100; *v*/*v*). Extracted proteins were separated by SDS-PAGE and transferred to nitrocellulose membranes. The membranes were blocked with 5 % non-fat dry milk in tris-buffered saline and tween 20 (TBST) and subsequently incubated with rabbit anti- neuronal nitric oxide synthase (nNOS) or rabbit anti-MPO antibodies (Santa Cruz Biotechnology, Inc., Dallas, TX; 1:200) overnight at 4 °C on a rocking platform. After washing, the membranes were incubated with peroxidase-conjugated anti-rabbit IgG (Jackson; 1:5000) and protein bands were detected by electrochemiluminescence (ECL) (Life Technologies, Grand Island, NY).

### Statistical analysis

Data were expressed as means ± SEM. Neurological scores were analyzed by nonparametric Mann-Whitney test. Other statistical analyses were performed using *t* test or one-way ANOVA with the appropriate post hoc test for multiple comparisons. A *p* value of <0.05 was considered statistically significant.

## Results and discussion

### KYC significantly reduced neurological severity scores and infarct size in MCAO mice

**Fig. 1:**
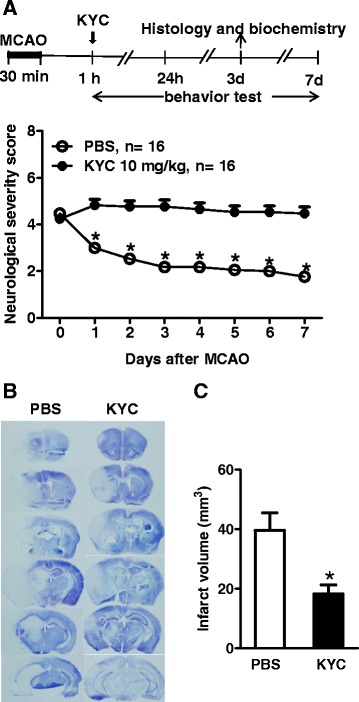
KYC significantly reduced neurological deficit and brain infarct size in mice subjected to MCAO. **a** Effects of KYC on neurological severity scores during 7 days after MCAO. *Top panel*: Experimental scheme; KYC (10 mg/kg/day) or PBS via i.p. was administrated starting 1 h after reperfusion. *Bottom panel*: Neurological severity scores of mice after MCAO (*p < 0.05, KYC vs. PBS group at same time interval, nonparametric Mann-Whitney test); **b** Effect of KYC on infarct size in mice 3 days after MCAO. Images of brain coronal sections (20-μm thick, 1-mm interval from rostral to caudal) by cresyl violet stain were shown; **c** Infarct volume (*p < 0.05, KYC vs. PBS group, *n* = 5/group, *t* test)

We treated MCAO mice with KYC for 7 days and determined the effects of KYC on neurological severity scores of mice after MCAO (Scheme, Fig. 1a, *top panel)*. PBS-treated MCAO mice group had a neurological severity score of 4.3 1 h after reperfusion which increased to 4.8 after 1 day, then slowly decreased to 4.4 by day 7 (Fig. 1a, *bottom panel*). However, the KYC-treated group (10 mg/kg/day, started 1 h after reperfusion) had an initial score of 4.5 at 1 h which decreased to 3.0 1 day after the initial KYC treatment and continued to decrease to 1.7 by day 7 with continued KYC treatment (Fig. 1a, *bottom panel*). As KYC is a specific inhibitor of MPO oxidant generation, these data clearly indicate that MPO-mediated oxidative stress plays a causal role in brain injury and neurological deficits after stroke. To answer the question whether KYC protects brains from ischemic injury before neutrophil recruitment, mice were treated with KYC 1 h before MCAO. Our study showed that KYC pre-treatment did not reduce neurological severity scores at 1 h post MCAO (data not shown). These data agree with previous studies [23, 39] showing that MPO was only detected in the brain several hours after reperfusion and that KYC specifically inhibits MPO activity in vivo.

Next, we investigated if KYC (10 mg/kg/day) reduces infarct size in mice 3 days after MCAO. Coronal sections in Fig. 1b showed that I/R injury induces a large infarct (absence of blue staining) (*left panel*) [36]. KYC treatment reduces the size of the infarct by 50 % (*p* < 0.05) (Fig. 1b, *right panel* and Fig. 1c). Taken together, these data provide a direct link between MPO activity to the damaged brain after stroke and that inhibiting MPO activity is an effective therapy for preserving brain tissue.

### KYC significantly protected BBB function and decreased neutrophil infiltration

**Fig. 2:**
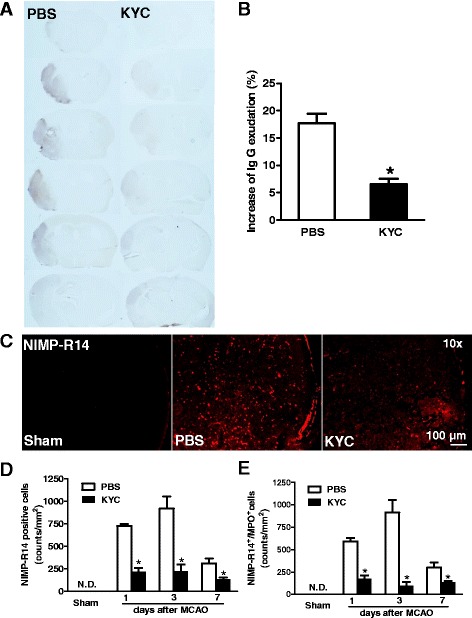
Effects of KYC on IgG extravasation and neutrophil infiltration in the brain of MCAO mice. MCAO mice were treated with KYC (10 mg/kg/d) or PBS via i.p. starting 1 h after MCAO. Brain tissues were harvested 3 days after MCAO. **a** Gross photographs of endogenous IgG extravasation in the brain of MCAO mice. Brain coronal sections from rostral to caudal in 1-mm interval were immunostained using an anti-mouse IgG (brown); **b** Mean gray value of IgG exudation (*p < 0.05, KYC vs. PBS group, *t* test); **c** Neutrophil infiltration in brain cortex ischemic core. Frozen sections were immunostained with anti-NIMP-R14 antibody (marker for neutrophil); **d** Counts of NIMP-R14^+^ cells in the brain sections of mice 1, 3, and 7 days after MCAO; **e** Counts of NIMP-R14^+^/MPO^+^ cells in the brain sections of mice 1, 3, and 7 days after MCAO (*n* = 4/group, *p* < 0.05, KYC vs. PBS group, *t* test)

BBB dysfunction is a hallmark feature of stroke. One of the ways to assess BBB function in vivo is to stain for tissue IgG, which cannot enter the brain unless BBB function is compromised [38]. Figure 2a (*left panel*) shows notable IgG extravasation in the ischemic brain hemisphere of the mouse 3 days after MCAO, which was notably reduced in MCAO mice treated with KYC (*p* < 0.05) (Fig. 2a*right panel* and 2b). Neutrophil infiltration is another hallmark of stroke. Figure 2c shows that MCAO induced significant increases in neutrophil infiltration in the ischemic hemisphere of the brain as compared to the brain of the sham mouse. KYC treatment significantly reduced neutrophil infiltration. Figure 2d, e shows the numbers of cell counts for neutrophils (NIMP-R14^+^) and colocalization of MPO and neutrophils (Additional file 1: Figure S3A). Our results showed that both neutrophil infiltration (Fig. 2d) and MPO^+^ neutrophils (Fig. 2e) were increased in MCAO brain 24 h after MCAO and peaked at the third day. The number of neutrophils was decreased at the seventh day after MCAO. KYC treatment significantly reduced the number of neutrophils and MPO^+^ neutrophils in MCAO mice from day 1 to day 7 (*p* < 0.05) (Fig. 2d, e). These data indicate that MPO participates in increasing BBB leakage and that inhibiting MPO activity restores BBB function and decreases neutrophil infiltration.

### KYC significantly reduced microglia/macrophage activation and neuron loss in MCAO mice

**Fig. 3:**
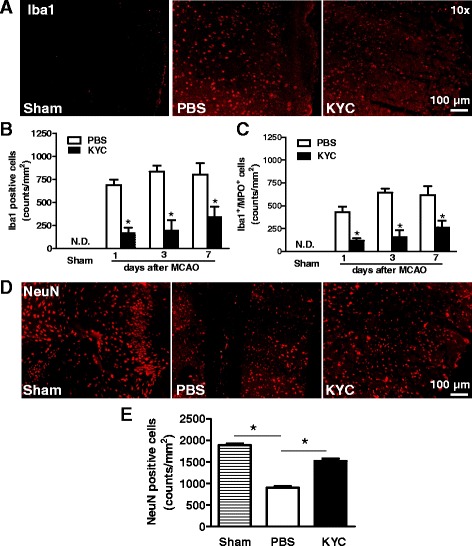
Effects of KYC on microglia activation and loss neuron in the brain of mice. MCAO mice were prepared as described in Fig. 2. **a** Images of Iba1^+^ in brain cortex ischemic core 3 days after MCAO; **b** Counts of Iba1^+^ cells in the brain sections of mice 1, 3, and 7 days after MCAO. **c** Counts of Iba1^+^/MPO^+^cells in the brain sections of mice 1, 3, and 7 days after MCAO. All data represented *n* = 4 (*p* < 0.05, KYC vs. PBS group, *t* test). **d** Images of immunostaining of neuron (NeuN) in brain cortex ischemic core 3 days after MCAO; **e** Counts of NeuN positive cells (*n* = 5/group, *p < 0.05, one-way ANOVA with Bonferroni test)

Immunofluorescent staining of brain sections showed significant increases in Iba1^+^ cells (Fig. 3a–c, Additional file 1: Figure S3B) and reductions in the number of neurons in the brains of mice 3 days after MCAO (Fig. 3d, e). Iba1^+^ cells and MPO expressing Iba1^+^ cells were increased starting on day 1 and continued to increase through day 7. Treating mice with KYC significantly reduced both Iba1^+^ cells and MPO expressing Iba1^+^ cells, suggesting that MPO expression in Iba1^+^ cells may be regulated by MPO activity. KYC (10 mg/kg/d) treatment also significantly reduced neuronal cell death (Fig. 3d, e, *p* < 0.05). These data indicate that MPO-mediated oxidative stress plays important roles in microglia/macrophage activation and neuronal cell death. However, KYC treatment has no effect on the numbers of glial fibrillary acidic protein (GFAP)-positive astrocytes even though MCAO increased GFAP-positive astrocytes (Additional file 1: Figure S1).

### KYC decreased apoptosis and cell injury in the brains of MCAO mice

**Fig. 4:**
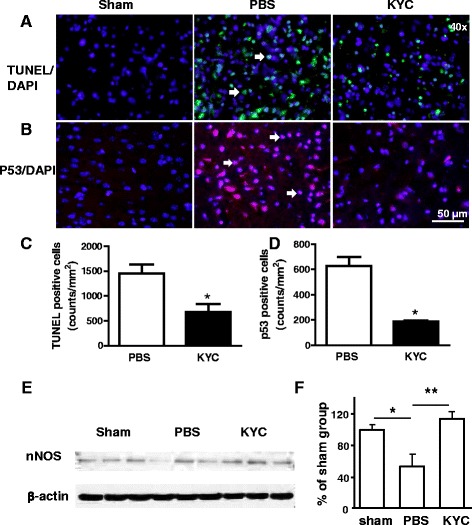
Effects of KYC on apoptosis in the brain of mice 3 days after MCAO. MCAO mice were prepared as described in Fig. 2. **a** Images of TUNEL assay in the cortex ischemic core areas; **b** Images of p53 immunostaining in brain cortex ischemic core; *blue*: DAPI, *red*: p53, and *purple*: p53 + DAPI, as *arrows* indicated. **c** Counts of TUNEL positive cells (*n* = 5/group, *p < 0.05, KYC vs. PBS group, *t* test). **d** Counts of p53 positive cells (*n* = 4/group, *p < 0.05, KYC vs. PBS group, *t* test). **e** Western blot of nNOS in MCAO brains; **f** Density analysis of nNOS western blot. The data shown is the ratio of nNOS density to β-actin density. The data for sham group is normalized to 100 % (*n* = 6/group, one-way ANOVA with Bonferroni test, *p < 0.05 and **p < 0.01)

To investigate if inhibition of MPO reduces apoptosis in MCAO brain, we assessed apoptosis in brain sections using the TUNEL assay. Our results showed that the number of TUNEL positive cells was increased in the brain 3 days after MCAO (Fig. 4a), whereas KYC treatment reduced the number of TUNEL positive cells by 50 % (Fig. 4a, c, *p* < 0.05). Tumor protein p53 (p53) is a key protein for cell response to various stress induced injuries. When cell injury occurs after stress, p53 is expressed and nuclear translocated, which leads to activating the repair of damaged cells, or to trigger apoptosis in injured cells where DNA damage is irreparable [40]. Since TUNEL assay may not be absolutely specific for apoptosis [41, 42], we determined p53 expression in cell nuclei. We found that p53 accumulation in nucleus in the brain of MCAO mice was significantly increased and KYC treatment reduced the p53 accumulation by 70 % (Fig. 4b, d, *p* < 0.05). Further study showed that 86.3 ± 0.3 % p53^+^ cells were colocalized with TUNEL^+^ cells in the sections of the brain from MCAO mice. Taken together, our results strongly suggested that KYC protects CNS cells from MPO-mediated apoptosis. Additional evidence that KYC reduced brain injury in MCAO mice comes from western blots of nNOS in brains (Fig. 4e, f) [43]. nNOS western blots show that MACO significantly reduced nNOS levels 3 days after MCAO (Fig. 4f, *p* < 0.05) and that KYC treatment markedly increased nNOS levels in the brains of MCAO mice (Fig. 4f, *p* < 0.01).

### KYC reduced MPO in the brains of MCAO mice

**Fig. 5:**
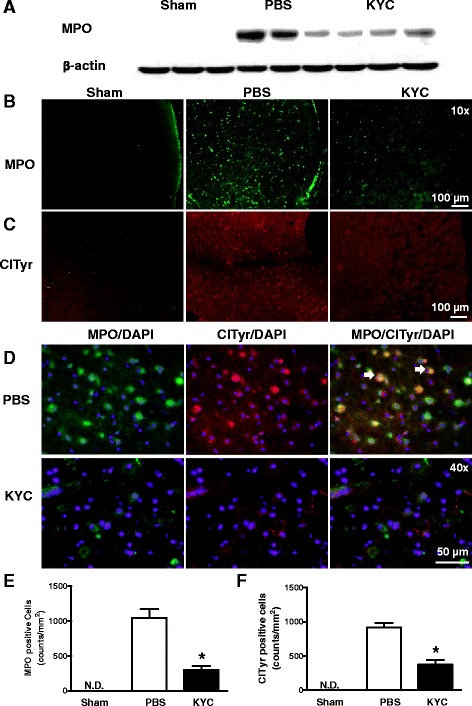
Effects of KYC on MPO and ClTyr expression in the brain of mice 3 days after MCAO. MCAO mice were prepared as described in Fig. 2. **a** Western blot of MPO in brain tissues of MCAO mice; **b** Images of MPO immunostaining in cortex ischemic core areas; **c** ClTyr immunostaining in the cortex ischemic core areas; **d** Images of colocalization of MPO and ClTyr in brain cortex ischemic core; *red*: ClTyr, *green*: MPO, and *yellow*: ClTyr + MPO, as arrows indicated. All images here are the representative images of five repeat samples. **e** Counts of MPO positive cells (*n* = 5/group, *p < 0.05, KYC vs. PBS group, *t* test). **f** Counts of ClTyr positive cells (*n* = 5/group, *p < 0.05, KYC vs. PBS group, *t* test)

To investigate if KYC also reduces the amount of MPO in the brains of MCAO mice, we analyzed MPO levels in the brains of mice 3 days after MCAO using western blot. Our data showed that MCAO induced a large increase of MPO levels in brain tissues (Fig. 5a) that was decreased in the brains of KYC-treated MCAO mice (Fig. 5a). Immunohistological studies also showed marked increases in MPO in brain sections from PBS-treated MCAO mice, which was significantly reduced by KYC treatment (Fig. 5b, e). Further analyses of MCAO tissues from day 1, 3, and 7 showed that both MPO activity and MPO immunostaining were increased in MCAO brain and peaked at third day after MCAO (Additional file 1: Figure S2A and S2B).

To confirm that KYC reduces MPO-dependent damage by inhibiting MPO activity, we probed the brain sections for ClTyr, a fingerprint biomarker of MPO activity. Figure 5c shows representative immunofluorescent stains of sham, PBS- and KYC-treated MCAO brain sections. Compared to sham, PBS-treated brains had marked increase in ClTyr immunostaining. In contrast, ClTyr formation was significantly reduced in KYC-treated MCAO mice (~65 %, Fig. 5c, f). Moreover, double immunofluorescent staining revealed ClTyr colocalized with MPO (orange-yellow) in the brains of MCAO mice and that KYC treatment essentially ablated colocalization (Fig. 5d). These data indicate KYC effectively inhibits MPO activity and MPO generation of toxic oxidants in MCAO brains.

### KYC reduced NO_2_Tyr and 4-HNE in MCAO mice

**Fig. 6:**
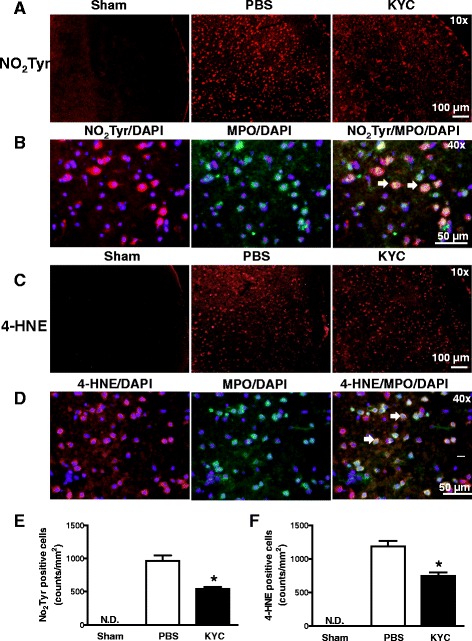
Effects of KYC on NO_2_Tyr and 4-HNE expression in the brain of mice 3 days after MCAO. MCAO mice were prepared as described in Fig. 2. **a** Images of NO_2_Tyr immunostaining in the cortex ischemic core areas; **b** Images of colocalization of MPO and NO_2_Tyr in brain cortex ischemic core; *red*: NO_2_Tyr, *green*: MPO, and *yellow*: NO_2_Tyr + MPO, as *arrows* indicated; **c** 4-HNE immunostaining in the cortex ischemic core areas; **d** Images of colocalization of 4-HNE and MPO in brain cortex ischemic core (*red*: 4-HNE, *green*: MPO, and *yellow*: 4-HNE + MPO, as *arrows* indicated). All images are the representative images of five repeat samples. **e** Counts of NO_2_Tyr positive cells (*n* = 5, * *p* < 0.05, KYC vs. PBS group, *t* test). **f** Counts of 4-HNE positive cells (*n* = 5, * *p* < 0.05, KYC vs. PBS group, *t* test)

MPO is known to generate a wide variety of free radicals that form stable adducts during oxidation of protein and lipids such as NO_2_Tyr and 4-HNE. To determine if MPO increases the formation of these stable oxidation products, sections of the brains were immunostained with antibodies that were specific for NO_2_Tyr and 4-HNE. Figure 6a shows that NO_2_Tyr immunostaining was markedly increased in the brains of mice 3 days after MCAO. KYC treatment of MCAO mice dramatically reduced brain NO_2_Tyr staining (Fig. 6e). Figure 6b shows that NO_2_Tyr co-localizes with MPO in MCAO brains (orange-yellow). These data indicate that MPO plays a major role in nitrosative damage in the brains of MCAO mice. 4-NHE was also increased in MCAO mice, which was significantly reduced with KYC treatment (Fig. 6c, f). Moreover, 4-HNE co-localizes with MPO in the brains of MCAO mice (Fig. 6d). Together, the data demonstrate that MPO-dependent oxidative stress significantly contributes to brain damage in MCAO mice.

This report shows that KYC, a novel competitive inhibitor of MPO, reduces oxidative damage to brains in an established murine model of stroke. In summary, our studies show that the benefits of targeting MPO-dependent oxidative stress are reductions in neurological severity scores, infarct size, BBB leakage, neutrophil infiltration, microglia/macrophage activation, neuronal loss, and apoptosis in the core of the lesions. Here, we provide direct evidence showing that KYC specifically inhibits MPO activity in MCAO mice based on (1) the fact that KYC decreases ClTyr formation, a molecular fingerprint of MPO oxidative activity and (2) the fact that ClTyr colocalization with MPO confirms that KYC targets MPO activity as we reported earlier [33]. Data here demonstrate that MPO-mediated oxidative stress plays a causal role in the mechanisms driving brain injury after stroke.

It is important to note that KYC did not reduce neurological scores after 1-h reperfusion when it was used to pretreat MCAO mice. The reason for this result is that MPO was not present during the period of ischemia and early reperfusion after stroke. Previous reports have shown that the increase of MPO in stroke occurs 6–12 h after ischemic injury [23, 39]. Thus, data here agree with our previous report showing that KYC specifically targets MPO in an EAE model of multiple sclerosis [30]. Our study showed that inhibiting MPO activity significantly reduced MPO activity and MPO protein in MCAO brain (Fig. 5a, b and Additional file 1: Figure S2) which is consistent with our published report showing that KYC treatment reduces the amount of MPO in the CNS of EAE mice [30]. The reason for such a change might be due to a decrease of neutrophil infiltration as well as microglia/macrophage activation. We found that MPO-dependent oxidative stress plays a major role in damaging BBB in MCAO mice during reperfusion (Fig. 2a) [30]. Inhibition of MPO reduced BBB leakage, which can also decrease neutrophil infiltration (Fig. 2c–e) as well as decrease the amount of MPO that can enter damaged MCAO brains. Another way KYC may decrease MPO in MCAO brains is inhibiting MPO activity, which reduces Iba^+^/MPO^+^ cells (Fig. 3a–c). These data are consistent with data from others who showed that MPO activity induces activated microglial cells/macrophages in MCAO brains to express MPO [44]. Accordingly, both a decrease in neutrophil infiltration and a reduction in microglia/macrophage activation by KYC treatment could contribute to significant reductions in MPO in the brains of MCAO mice.

Although there is overwhelming evidence for oxidative stress playing a role in neuronal damage induced by stroke, clinical trials have failed to show that antioxidants improve outcomes in stroke patients [45, 46]. Moreover, some large clinical trials have concluded that antioxidant supplementation could even be harmful [13–15]. Several reasons have been proposed to explain why previous antioxidant clinical trials did not work [13–15]. Generally, the reaction rates between antioxidants and ROS are slower than the rates between biological molecules and ROS. Secondly, although antioxidants scavenge free radicals in vivo, the oxidized products of antioxidants can become harmful free radicals that also cause tissue damage. Moreover, recent studies have found that ROS play important roles as signaling molecules under normal physiological conditions. ROS become detrimental in many diseases only when there is an over-production of ROS that causes oxidative stress in vivo. Accordingly, using non-specific antioxidants could interfere with the beneficial effects of ROS required for normal physiological function. To overcome problems associated with antioxidant therapy, it was suggested that inhibitors targeting oxidant formation from specific sources is what is needed to reduce oxidative stress in stroke [47]. Here, using an established MCAO murine model of stroke, we show that KYC, an MPO-specific inhibitor, effectively reduces brain injury after stroke. Indeed, just 3 days of KYC treatment was sufficient to significantly reduce infarct size, BBB dysfunction, neutrophil infiltration, and neuronal cell death. Our results strongly support the idea that targeting of specific oxidative sources, in this case, MPO with KYC, is an effective therapeutic strategy for treating stroke patients.

Previous studies have shown that oxidation products were increased in stroke patients and animal models [6, 9, 10, 24, 48–56]. It is well known that nNOS is activated and iNOS expression is upregulated after I/R treatment [57–59]. NO_2_Tyr was found to be increased in both the core and surrounding penumbra after ischemia and even further increased during reperfusion in animal models of stroke [6, 50, 51]. The formation of NO_2_Tyr in stroke has been largely attributed to peroxynitrite formation [58, 60]. However, our experiments show that NO_2_Tyr formation is significantly reduced by KYC (Fig. 6). The fact that NO_2_Tyr can be co-localized with MPO in MCAO mice brains suggests that MPO-mediated nitration is a major pathway for NO_2_Tyr formation after stroke. Lipid peroxidation products are also significantly increased in the plasma of stroke patients (14–19). Our data show that KYC reduced 4-HNE, a stable lipid peroxidation product, in the brains of MCAO mice (Fig. 6). Such data indicate that MPO-dependent oxidation may also increase the generation of lipid peroxidation products. Taken together, this study clearly suggests that MPO-dependent oxidative stress plays an important role in oxidative injury of the brain after stroke and that inhibiting MPO oxidant production could be an effective therapeutic strategy for reducing oxidative stress and decreasing brain injury in stroke patients.

## Conclusions

KYC, a specific competitive inhibitor of MPO activity, effectively inhibits MPO generation of toxic oxidants in vivo. By doing so, KYC reduces neuronal damage and preserves brain tissue and neurological function in the stroked brain. On the basis of these findings, we conclude that MPO-mediated oxidative stress plays an important role in the mechanisms by which stroke induces oxidative injury to the brain and accordingly KYC should be a highly effective therapeutic agent for treating patients with stroke.

## Additional file

Additional file 1: Figure S1. Effect of KYC on astrocytes activation in the brain of mice 3 days after MCAO. Mice were treated with PBS or KYC (10 mg/kg/d) via i.p. starting 1 h after MCAO. A. Images of immunostaining of astrocytes (GFAP) in brain cortex ischemic core; B. Counts of GFAP-positive cells. Figure S2. The changes of MPO activity and protein in the brain of mice after MCAO. MCAO mice were prepared as described in Fig. 2 and brain tissues were harvested 1, 3, and 7 days after MCAO. A. MPO activity. MPO in samples was extracted as follows [61]: Brain tissues were homogenized in 0.1 M potassium phosphate buffer pH 6.0 containing 0.5 % cetyltrimethylammonium bromide. The samples were ultrasonicated for 30 s and went through three freeze-thaw cycles. Finally, the samples were centrifuged at 15,000g for 15 min and supernatants were saved for MPO activity assay. MPO activity assay was performed based on our previously published method with modifications [30]: Briefly, extracted samples were mixed with 50 mM sodium phosphate buffer pH 5.4 containing 100 μM diethylenetriaminepentaacetic acid, 10 mM NaNO_2_, 100 μM hydrogen peroxide, and 50 μM Amplex Ultra Red. After incubation at 37 °C for 15 min, the fluorescence intensity was measured at ex = 540 nm, em = 590 nm. All data represents *n* = 3/group. Sham were from day 1, 3, 7 (*n* = 3/group). (**p* < 0.05 and ****p* < 0.001, KYC vs. PBS group, *t* test). B. Counts of MPO^+^ cells in cortex ischemic core area. (*n* = 4/group; **p* < 0.05, KYC vs. PBS, *t* test). Figure S3. Effects of KYC on the expression of MPO in NIMP-R14 and Iba1 positive cells in the cortex ischemic core areas of mice after MCAO. MCAO mice were prepared as described in Fig. 2 and brain tissues were harvested 1, 3, and 7 days after MCAO. A. Images of immunostaining of MPO^+^ and NIMP-R14^+^ cells (green: MPO, red: NIMP-R14, and yellow: MPO + NIMP-R14, as arrows indicated). B. Images of immunostaining of MPO^+^ and Iba1^+^ cells (green: MPO, red: Iba1, and yellow: MPO + Iba1, as arrows indicated). All images here are the representative images of four repeat samples. Figure S4. Colocalization of p53 immunostaining with TUNEL positive cells in brain cortex ischemic core of mice 3 days after MCAO. Red: p53, green: TUNEL, yellow: p53 + TUNEL, as arrows indicated. Images represent three repeats. (PDF 586 kb)

## Abbreviations

4-HNE: 4-hydroxynonenal
ABAH: 4-aminobenzoic acid hydrazide
ANOVA: analysis of variance
BBB: blood-brain-barrier
ClTyr: chlorotyrosine
CNS: central nervous system
DAPI: 4′,6-diamidino-2-phenylindole
DNA: deoxyribonucleic acid
EAE: experimental allergic encephalomyelitis
ECL: electrochemiluminescence
EDTA: ethylenediaminetetraacetic acid
GFAP: glial fibrillary acidic protein
I/R: ischemia/reperfusion
Iba1: ionized calcium-binding adapter molecule 1
IgG: immunoglobulin G
KYC: *N*-acetyl lysyltyrosylcysteine amide
MCAO: middle cerebral artery occlusion
MDA: malondialdehyde
MPO: myeloperoxidase
NIH: National Institutes of Health
nNOS: neuronal nitric oxide synthase
NO2Tyr: nitrotyrosine
PBS: phosphate-buffered saline
RIPA: radio-immunoprecipitation assay
ROS: reactive oxygen species
RT: room temperature
SDS-PAGE: sodium dodecyl sulfate polyacrylamide gel electrophoresis
SEM: standard error of the mean
TBST: tris-buffered saline and tween 20
TdT: terminal deoxynucleotidyl transferase
TUNEL: terminal deoxynucleotidyl transferase-mediated dUTP-biotin nick end labeling
