# Erratum to: Inhibition of myeloperoxidase oxidant production by N-acetyl lysyltyrosylcysteine amide reduces brain damage in a murine model of stroke

**DOI:** 10.1186/s12974-016-0639-y

**Published:** 2016-06-27

**Authors:** Guoliang Yu, Ye Liang, Ziming Huang, Deron W. Jones, Kirkwood A. Pritchard, Hao Zhang

**Affiliations:** Division of Pediatric Surgery, Department of Surgery, Medical College of Wisconsin, 8701 Watertown Plank Rd, Milwaukee, 53226 WI USA; Department of Breast Surgery, Maternal and Child Health Hospital of Hubei Province, 745 WuLuo Road, Hongshan District, Province 430070, Wuhan City, Hubei China

## Erratum

After publication of this work [1], it was noted that there was an error within Fig. 1. Within the graph of Fig. 1a, the labels for PBS and KYC were inadvertently reversed. The open circles should represent KYC, whereas the filled circles should represent PBS. Figure 1 has been corrected in the original article and is also included correctly below.

**Fig. 1:**
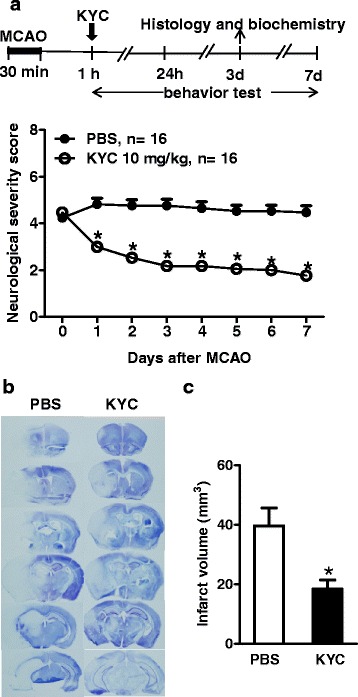
KYC significantly reduced neurological deficit and brain infarct size in mice subjected to MCAO. **a** Effects of KYC on neurological severity scores during 7 days after MCAO. *Top panel*: Experimental scheme; KYC (10 mg/kg/day) or PBS via i.p. was administrated starting 1 h after reperfusion. *Bottom panel*: Neurological severity scores of mice after MCAO (*p < 0.05, KYC vs. PBS group at same time interval, nonparametric Mann–Whitney test); **b** Effect of KYC on infarct size in mice 3 days after MCAO. Images of brain coronal sections (20-μm thick, 1-mm interval from rostral to caudal) by cresyl violet stain were shown; c Infarct volume (*p < 0.05, KYC vs. PBS group, *n* = 5/group, t test)
