# Supplementary figures and images for: Inhibition of myeloperoxidase oxidant production by N-acetyl lysyltyrosylcysteine amide reduces brain damage in a murine model of stroke
Source: J Neuroinflammation. 2016 May 24;13:119. doi: 10.1186/s12974-016-0583-x (PMC4879722; doi:10.1186/s12974-016-0583-x)

Suppl. Figure 1

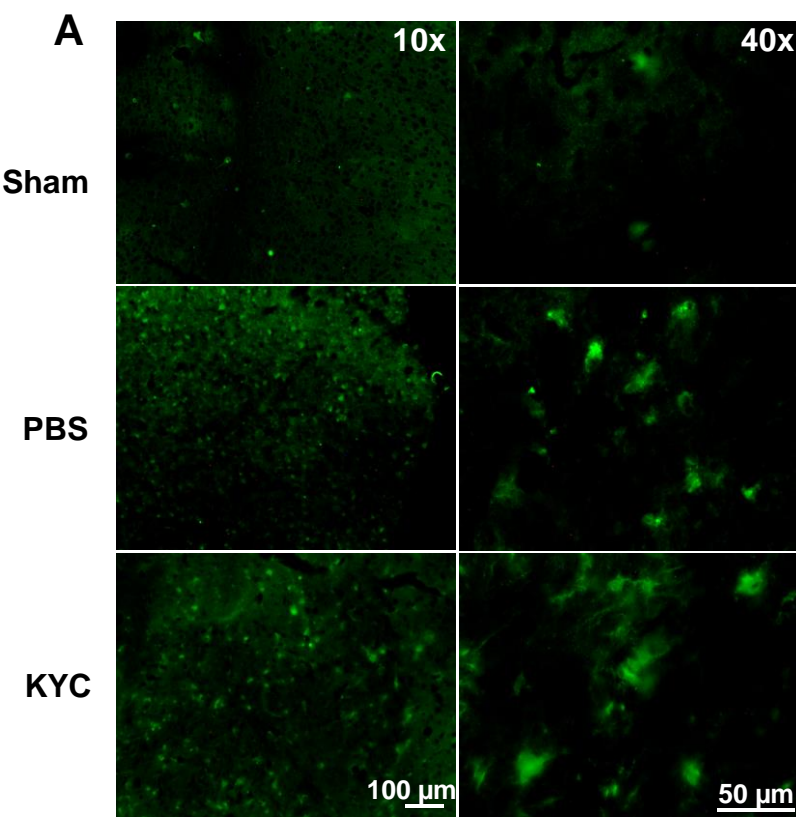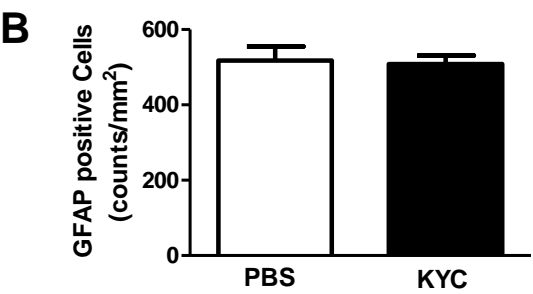

Suppl. Figure 2

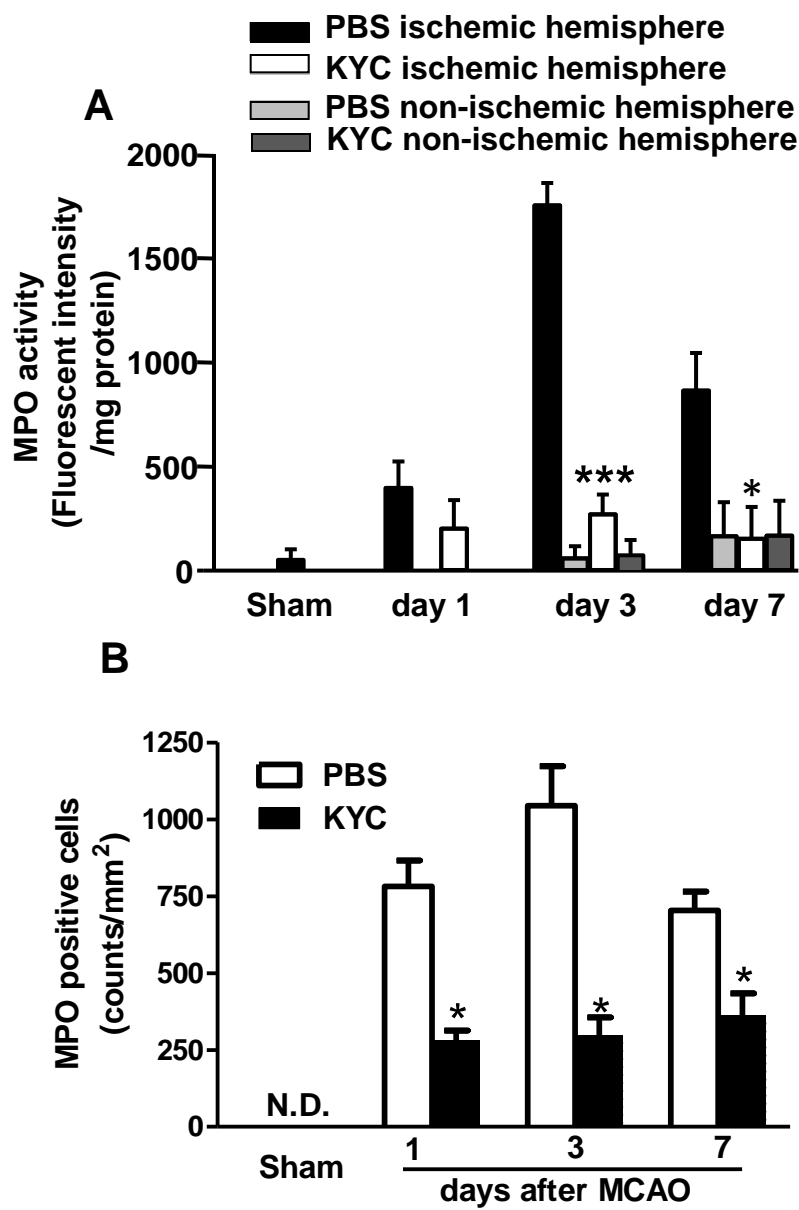

Suppl. Figure 3A

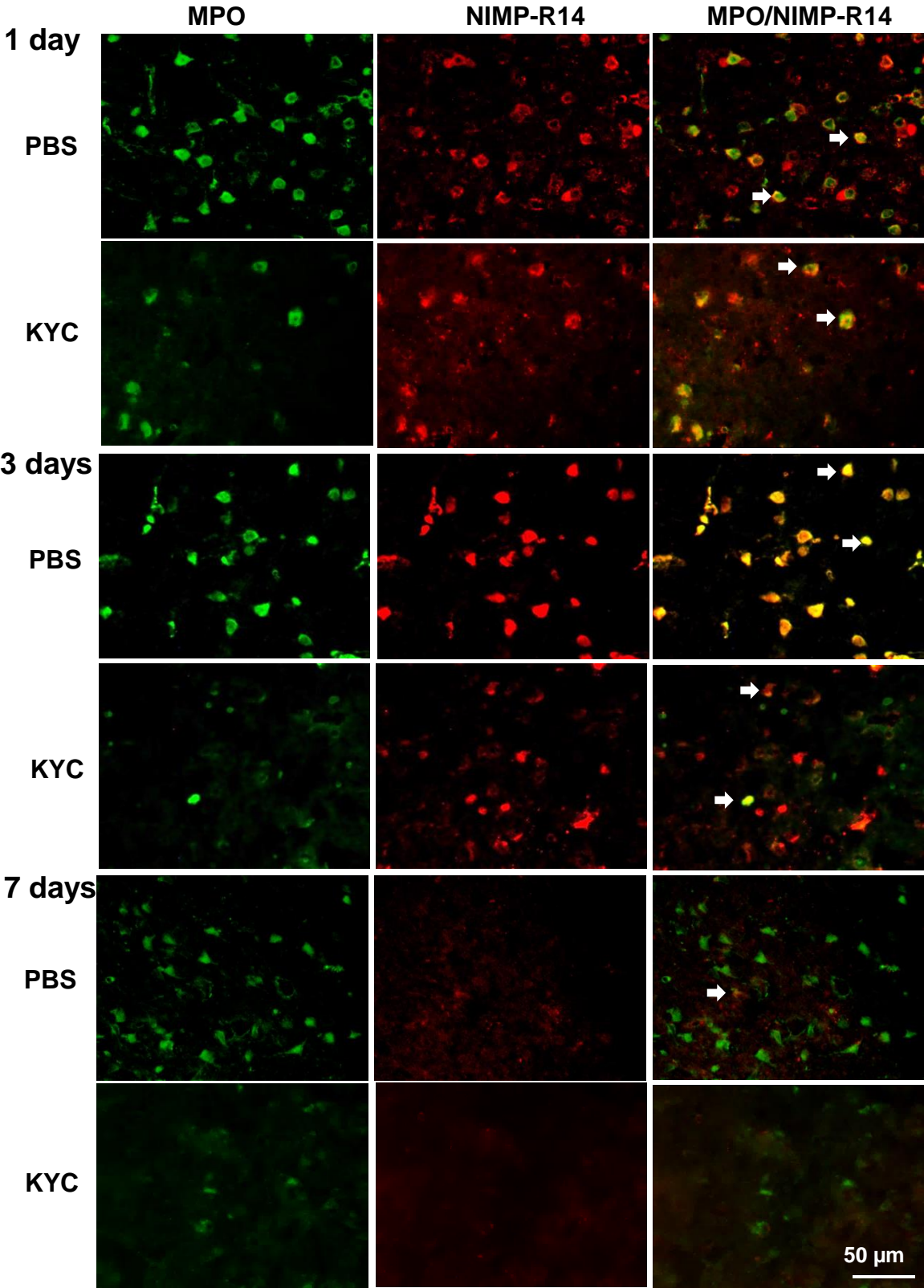

Suppl. Figure 3B

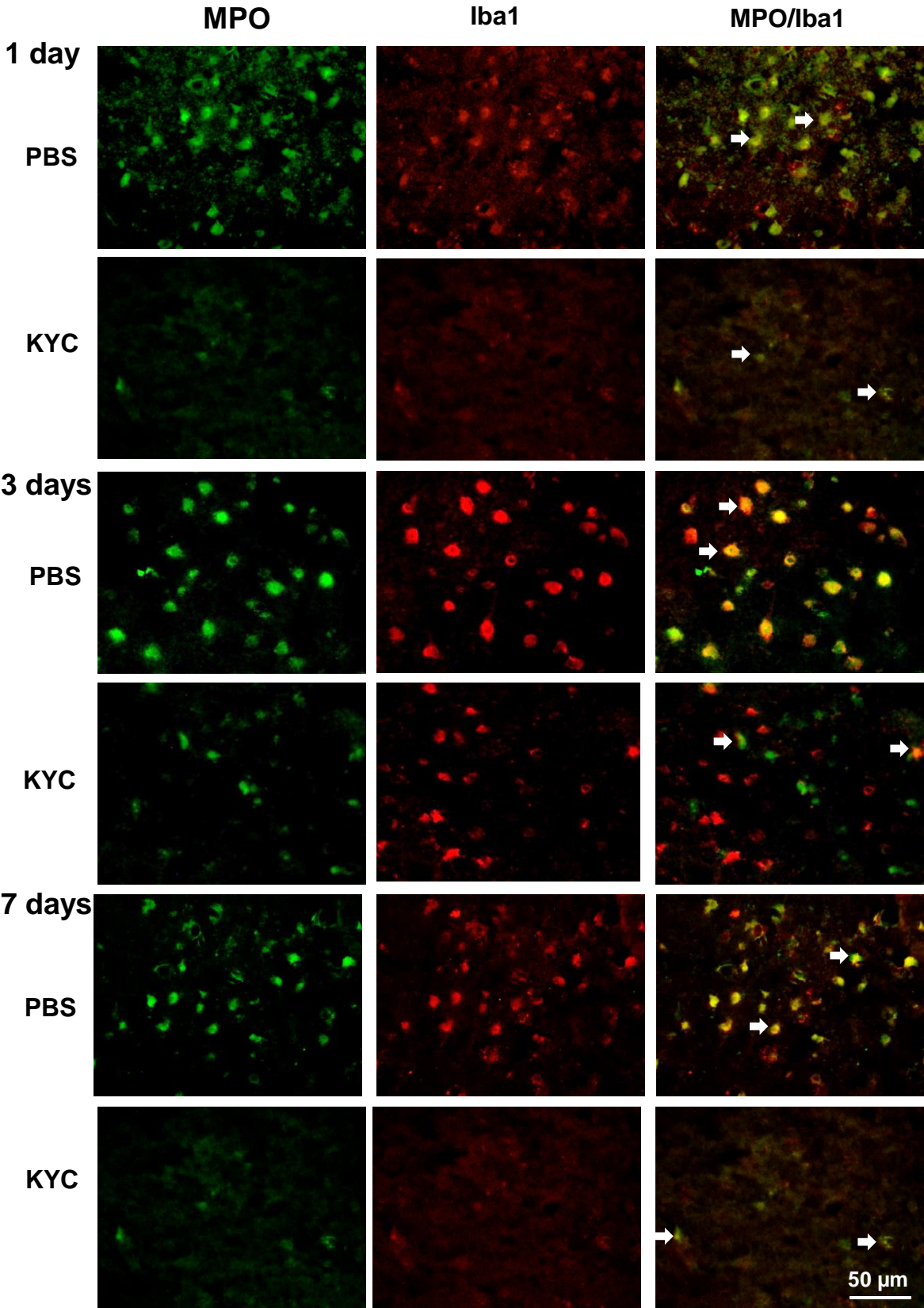

Suppl. Figure 4.

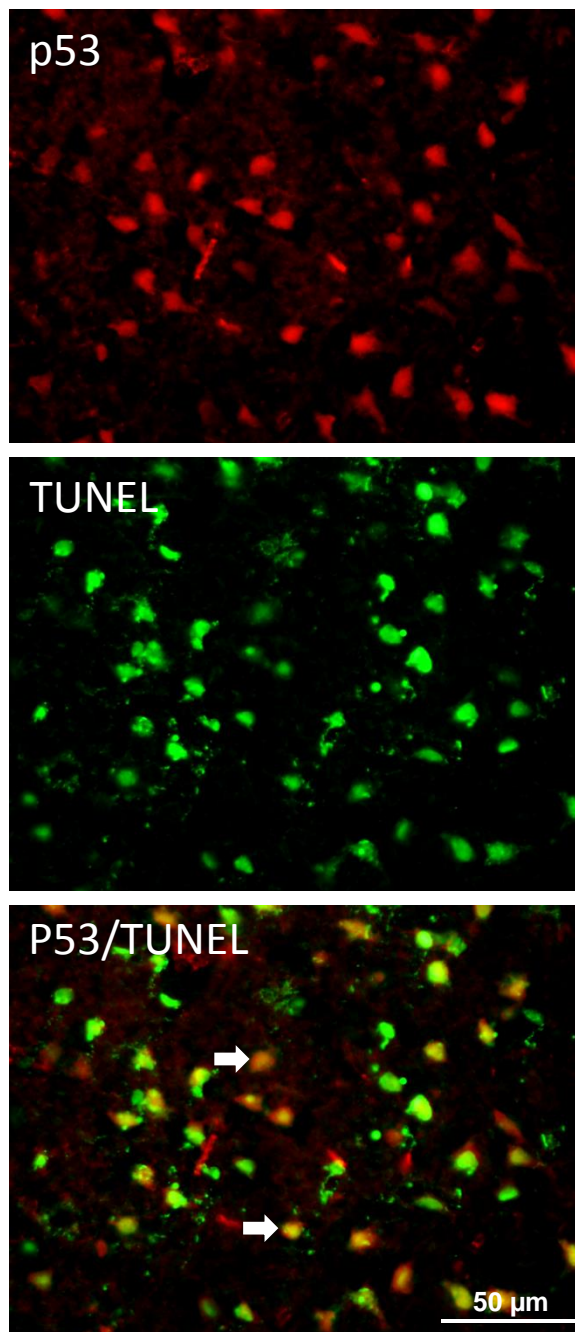

Supplement: Additional file 1: Figure S1. — Effect of KYC on astrocytes activation in the brain of mice 3 days after MCAO. Mice were treated with PBS or KYC (10 mg/kg/d) via i.p. starting 1 h after MCAO. A. Images of immunostaining of astrocytes (GFAP) in brain cortex ischemic core; B. Counts of GFAP-positive cells. Figure S2. The changes of MPO activity and protein in the brain of mice after MCAO. MCAO mice were prepared as described in Fig. 2 and brain tissues were harvested 1, 3, and 7 days after MCAO. A. MPO activity. MPO in samples was extracted as follows [61]: Brain tissues were homogenized in 0.1 M potassium phosphate buffer pH 6.0 containing 0.5 % cetyltrimethylammonium bromide. The samples were ultrasonicated for 30 s and went through three freeze-thaw cycles. Finally, the samples were centrifuged at 15,000g for 15 min and supernatants were saved for MPO activity assay. MPO activity assay was performed based on our previously published method with modifications [30]: Briefly, extracted samples were mixed with 50 mM sodium phosphate buffer pH 5.4 containing 100 μM diethylenetriaminepentaacetic acid, 10 mM NaNO2, 100 μM hydrogen peroxide, and 50 μM Amplex Ultra Red. After incubation at 37 °C for 15 min, the fluorescence intensity was measured at ex = 540 nm, em = 590 nm. All data represents n = 3/group. Sham were from day 1, 3, 7 (n = 3/group). (*p < 0.05 and ***p < 0.001, KYC vs. PBS group, t test). B. Counts of MPO+ cells in cortex ischemic core area. (n = 4/group; *p < 0.05, KYC vs. PBS, t test). Figure S3. Effects of KYC on the expression of MPO in NIMP-R14 and Iba1 positive cells in the cortex ischemic core areas of mice after MCAO. MCAO mice were prepared as described in Fig. 2 and brain tissues were harvested 1, 3, and 7 days after MCAO. A. Images of immunostaining of MPO+ and NIMP-R14+ cells (green: MPO, red: NIMP-R14, and yellow: MPO + NIMP-R14, as arrows indicated). B. Images of immunostaining of MPO+ and Iba1+ cells (green: MPO, red: Iba1, and yellow: MPO + Iba1, as arrows indicated). All i [file 12974_2016_583_MOESM1_ESM.pdf]
